# The Effect of Artificial Media and Temperature on the Growth and Development of the Honey Bee Brood Pathogen *Ascosphaera apis*

**DOI:** 10.3390/biology10050431

**Published:** 2021-05-12

**Authors:** Petr Mráz, Marian Hýbl, Marek Kopecký, Andrea Bohatá, Jana Konopická, Irena Hoštičková, Petr Konvalina, Jan Šipoš, Michael Rost, Vladislav Čurn

**Affiliations:** 1 Department of Genetics and Agricultural Biotechnology, Faculty of Agriculture, University of South Bohemia, Studentska 1668, 370 05 Ceske Budejovice, Czech Republic; konopj01@zf.jcu.cz (J.K.); jelini00@zf.jcu.cz (I.H.); rost@zf.jcu.cz (M.R.); curn@zf.jcu.cz (V.Č.); 2 Department of Zoology, Fisheries, Hydrobiology and Apiculture, Faculty of AgriSciences, Mendel University in Brno, Zemedelska 1, 613 00 Brno, Czech Republic; mario.eko@seznam.cz (M.H.); jan.sipos@mendelu.cz (J.Š.); 3 Department of Agroecosystems, Faculty of Agriculture, University of South Bohemia, Studentska 1668, 370 05 Ceske Budejovice, Czech Republic; mkopecky@zf.jcu.cz (M.K.); konvalina@zf.jcu.cz (P.K.); 4 Department of Crop Production, Faculty of Agriculture, University of South Bohemia, Studentska 1668, 370 05 Ceske Budejovice, Czech Republic; bohata@zf.jcu.cz; 5 Biology Centre of the Czech Academy of Sciences, Institute of Entomology, 370 05 Ceske Budejovice, Czech Republic

**Keywords:** *Apis mellifera*, chalkbrood, cultivation, culture media, sporulation

## Abstract

**Simple Summary:**

Chalkbrood is a worldwide spread honey bee brood disease caused by the fungal pathogen *Ascosphaera apis*. The disease is commonly treated with fungicides, but due to the accumulation of residues, these fungicides have been banned in many countries, including European Union countries. Since then, control of chalkbrood has been problematic. The disease is fatal to individual honey bee larvae and can cause significant losses in terms of both bee numbers and colony productivity, and can even in some cases lead to colony collapse. Owing to these reasons, in vitro fungus cultivation is necessary to properly understand its pathogenesis as well as life cycle for the possible future development of an efficient and environmentally friendly control method. Therefore, in this study, several artificial media and different temperatures were evaluated to see their impact on the growth and development of *A. apis*. Furthermore, one of the media was modified by the addition of crushed honey bee brood to simulate natural conditions. This medium was found to be the most suitable for fungus reproductive structure production. In addition, a biological pattern was found explaining the relationships between temperature and the size of the fungal reproductive structures.

**Abstract:**

*Ascosphaera apis* is a causative agent of chalkbrood, which is one of the most widespread honey bee diseases. In our experiments, the influence of several artificial media and cultivation under different temperatures was evaluated. Concretely, the radial growth of separated mating types was measured, reproductive structures in a Neubauer hemocytometer chamber were counted simultaneously, and the morphometry of spore cysts and spore balls was assessed. The complex set of experiments determined suitable cultivation conditions. A specific pattern between reproductive structure size and temperature was found. The optimal temperature for both mating types was 30 °C. SDA and YGPSA media are suitable for fast mycelial growth. Moreover, the effect of bee brood on fungus growth and development in vitro was investigated by modification of culture medium. The newly modified medium PDA-BB4 was most effective for the production of the reproductive structures. The result suggests that honey bee brood provides necessary nutrients for proper fungus development during in vitro cultivation. As there is no registered therapeutic agent against chalkbrood in most countries, including the European Union, the assessment of *A. apis* growth and development in different conditions could help to understand fungus pathogenesis and thus control chalkbrood disease.

## 1. Introduction

The western honey bee (*Apis mellifera*) is one of the most important pollinators of many agricultural crops as well as herbaceous plants [1,2,3]. However, a sudden large decline in the number of honey bees has been observed worldwide [4,5]. Especially in the United States of America, the number of bee colonies has decreased by over 50% since the 1940s [2,6,7]. Many factors are responsible for reducing bee colonies’ vitality and viability, including the application of acaricides, fungicides, and antibiotics [8], agrochemicals [9,10], malnutrition [11,12], inappropriate beekeeping practices or changes in habitat [5,13], and, especially, bee diseases [14,15]. One of the pathogens harming colonies is a widespread heterothallic fungus, *Ascosphaera apis* (Maasen ex Claussen) L.S. Olive and Spiltoir, which causes chalkbrood disease [16].

*Ascosphaera apis* (Ascomycota, Eurotiomycetes, Ascosphaerales) is a species closely adapted to honey bees and can only invade bee brood. It was widely accepted that *A. apis* spores cannot germinate on the larval cuticle, therefore, the infection of bee brood starts when the spores are ingested with the food by bee larvae during their feeding [14]. The mode of larval infection distinguishes this species from other entomopathogenic fungi belonging to the order Hypocreales, which are able to penetrate the insect cuticle without the need for ingestion [17,18]. For this reason, it is not possible to utilize many well-known methods that are used for studying common entomopathogenic fungi [19]. After ingestion, the spores of *A. apis* need to be exposed to higher CO_2_ concentrations to start germination [20]. In this case, CO_2_ is produced by the larval tissues in the gut. The optimal temperature for spore germination is 35 °C [21]. After their activation, spores become swollen and create germ tubes that grow into dichotomous hyphae. Mycelium then penetrates through the peritrophic membrane of bee larvae into the body cavity and grows to the posterior end, where it breaks the barrier. In the case of the presence of both mating types, it starts to create spore cysts (ascomata). Ascospores, which are the only infective units causing chalkbrood [22], are formed in spore balls (asci) and located in resistant cysts [16]. The spores contain two nuclei; the bigger one lies in the center and the second smaller one is situated near the end of the spore [23]. The three-layered spore wall is tough, containing chitin as its major component [23,24], which helps ascospores stay viable for many years [20,25,26].

The first clinical signs of the disease are dead larvae that are covered by a fluffy white mold and they are usually swollen. After some time, they shrink and turn black/gray or white, depending on the presence of reproductive structures [26,27,28]. At the end of the fungus development cycle, honey bee larvae become mummified. Chalkbrood can be easily recognized by visual detection of these mummified bee brood, known as chalkbrood mummies, on the bottom board of beehives, as well as in uncapped cells. However, De Jong [29] claims that a low infestation level of this disease (less than 12% infection) is not recognizable, because worker bees remove the infected bee brood.

Chalkbrood is considered to be a stress-related disease [27,30,31], which could be the reason for its increased occurrence in recent years [22,23], because honey bees are stressed by many factors. According to Vojvodic et al. [31], chalkbrood is a chronic disease because it persists in beehives for a long time and can break out at any time, depending on the conditions, which makes this disease more dangerous. Chalkbrood is also considered to be more prevalent in damp weather conditions [32,33,34], fluctuating temperatures [35], or if the bee colonies are excessively fed with sugar syrup [36]. Brood chilling also causes stress and leads to outbreaks of the disease [21,31,37,38]. This situation can occur during rapid colony growth due to a lot of bee brood in combs and a relatively small number of worker bees taking care of the brood and warming the space [29,38] up to a temperature close to the optimal bee brood temperature of 35 °C [27].

There are some indications that this fungus, which is one of the most contagious and destructive bee brood pathogens [14], may be increasing in occurrence [22]. There are also some locations, such as northern Thailand [39] and China [23], where this pathogen causes high bee mortality because the even less pathogen-susceptible *Apis cerana cerana* can be easily infected by *A. apis* [40]. This fact contributes to chalkbrood’s worldwide occurrence and adds to the importance of the disease. Although chalkbrood is fatal to individual bee larvae, it rarely causes colony losses [22]. However, in most cases, it causes significant losses of bee numbers and colony productivity [30,41], which makes it an economically important disease [27].

There is no registered therapeutic agent against chalkbrood disease [42] and it is difficult to involve or find any effective control agent [26,43] despite the promising results with plant essential oils recently [44,45]. Thus, for these reasons, it is necessary to understand the life cycle and pathogenesis of *A. apis*. From this point of view, the optimization of cultivation methods and the establishment of a stable and effective in vitro cultivation system with a high yield of ascospores are crucial.

Fungi have evolved several specialized strategies for overcoming insect defense mechanisms and reaching nutrients, including biotrophy (nutrition derived only from living cells, which ceases once the cell has died), necrotrophy (killing and utilization of dead tissues), and hemibiotrophy (initially biotrophic and then becoming necrotrophic). When they invade an appropriate kind of insect, the host provides them with the whole spectrum of nutrients they need [46]. Artificial media significantly influence the germination, growth, enzyme production, and thus also the virulence of entomopathogenic fungi [47,48]. Nutrition for fungi cultivated in vitro depends especially on the C/N ratio. Yeast extract and peptone have C/N ratios of 3.6:1 and 8:1, respectively, and represent different carbon and nitrogen sources [49]. According to Ibrahim et al.,2002 [47], nutrient-rich media such as SDA and YEA promote greater germination compared to nutrient-poor media. However, it can vary significantly depending on the species [49,50].

In the present study, commonly used artificial media as well as different temperatures were tested for the pathogen cultivation, with the aim of determining the optimal conditions for *A. apis* and verifying their influence on the reproductive structures’ yield and morphometry. Moreover, the effect of bee brood addition on fungus growth and sporulation was compared. These findings are necessary to understand fungus pathogenesis and occurrence in beehives and the biological mechanisms of reducing spore loads in nature.

## 2. Materials and Methods

### 2.1. Fungal Isolate

**Figure 1:**
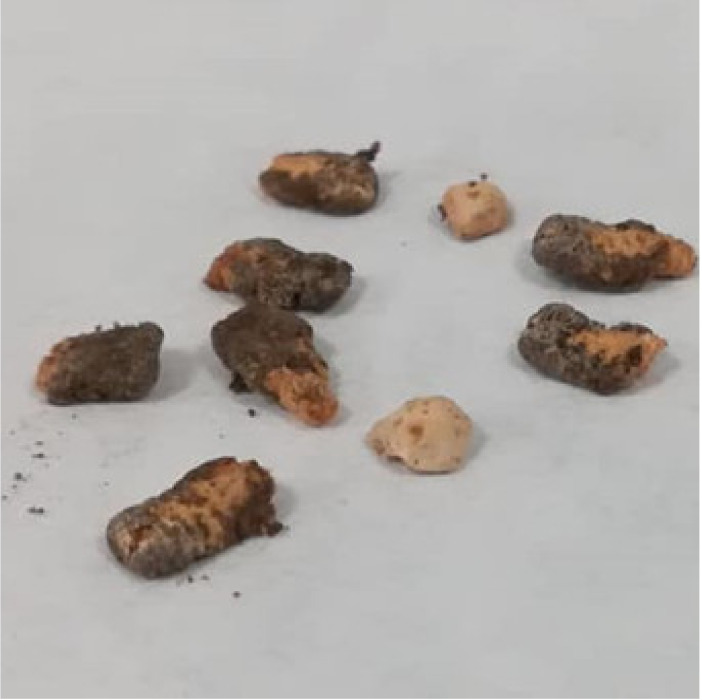
Mummified honey bee brood.

**Figure 2:**
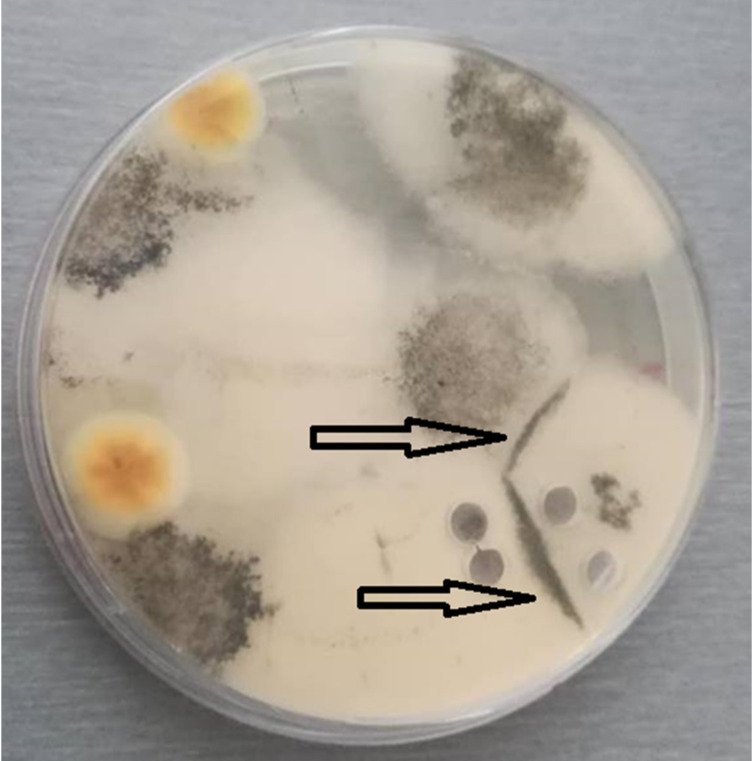
Separation of opposite mating types of *A. apis* by subcultivation. The mummified honey bee brood were crushed and subcultivated on PDA medium until black lines of reproductive structures appeared (shown by the two arrows). These lines indicated the presence of opposite mating types of *A. apis*. Mycelium was taken by a cork borer from that area.

**Figure 3:**
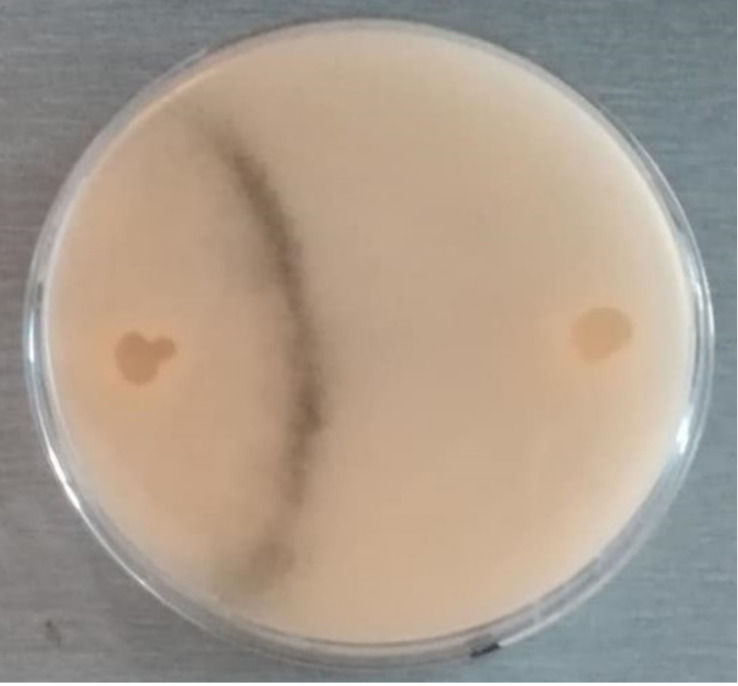
Pure cultures of opposite mating types of *A. apis* in dual test. Mycelia of opposite mating types were subjected to dual tests several times until pure cultures were established. The black line of reproductive structures is closer to the left side because of the slower growing (+) mating type.

**Figure 4:**
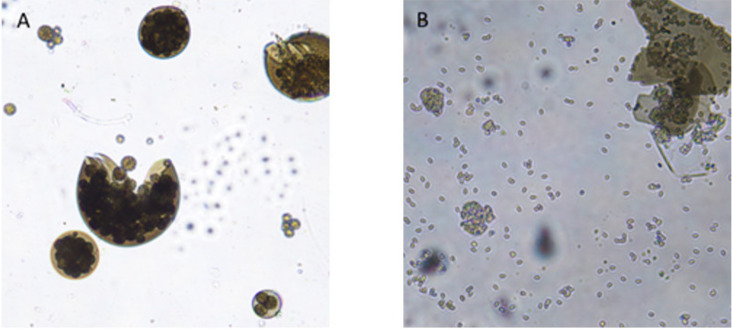
Spore cysts (**A**), spore balls (**A**), and ascospores (**B**). Reproductive structures of *A. apis* consist of spore cysts in which ascospores are formed in spore balls. If the cysts mature, they will open and ascospores can be released.

Dry mummified bee brood (Figure 1) were collected from an apiary in the South Bohemian Region, Czech Republic, from the bottom boards of infected colonies and stored in a refrigerator (at 5 °C) until use. The mummies were crushed and small pieces were placed on potato dextrose agar (PDA) for spore activation. The plates were incubated at 35 °C until the growth of mycelia and subsequent sporulation were observed. During that time, ascospores of this fungus were activated and the opposite mating types observed. Male (−) and female (+) mating types were separated by continuous subculturing (Figure 2) until pure cultures were obtained. The opposite mating types were verified by a dual test (Figure 3), when reproductive structures (Figure 4) in lines between mycelia of opposite mating types were developed.

Maternal cultures of both mating types were maintained separately on PDA at 30 °C. The fungus was identified by molecular techniques. PCR and sequencing of the ITS1-5.8S-ITS2 (ITS) region were carried out. Genomic DNA of *A. apis* was extracted and used as a template for PCR identification. The final volume was 20 µL, including 1 µL of template DNA, 10 µL PPP Master Mix (Top-Bio, Vestec, Czech Republic), 7 µL of PCR Ultra H_2_O (Top-Bio), and the specific ITS primers AscosFOR (TGTGTCTGTGCGGCTAGGTG) and AscosREV (GCTAGCCAGGGGGGAACTAA), 1 µL each at 10 µM [51]. PCR was performed under the following conditions: initial template denaturation at 95 °C for 2 min; 35 cycles at 95 °C for 30 s, 50 °C for 30 s, and 72 °C for 45 s, with a final 7 min extension at 72 °C. PCR products were electrophoretically separated on 1.5% agarose gel, visualized by a UV transilluminator (INGENIUS, Trigon-plus, SYNGENE, Cestlice, Czech Republic), purified using a gel extraction kit following the manufacturer’s instructions, and sent to SEQme (Dobris, Czech Republic) for data sequencing. Species identity was analyzed in the BLAST search tool available through the website of the NCBI [52] and confirmed by phylogenetic analysis. The ITS dataset used in this study was obtained from GenBank [53]. The *Aspergillus terreus* ITS sequence (accession number NR_149331) was used as an outgroup for the analysis. Sequence data were aligned using the CLUSTALw algorithm as implemented in Geneious 8.1.9. MrBayes was run for 100,000 generations with 50,000 sample points under default prior probability settings.

Seven standard artificial media, namely potato dextrose agar, (PDA), Sabouraud dextrose agar (SDA), malt extract agar (MEA), Czapek dox agar (CDA), potato dextrose agar with 0.4% yeast extract (PDAY), and yeast glucose starch agar (YGPSA) (all from HiMedia Laboratories Pvt. Ltd., Prague, Czech Republic), and Iso-Sensitest agar (Oxoid ISA) (Thermo Scientific™, Pardubice, Czech Republic) were used. Three modified media were prepared by the addition of crushed frozen drone bee brood to PDA before sterilization (PDA-BB4: 40 g/L PDA; PDA-BB8: 80 g/L PDA; PDA-BB12: 120 g/L PDA). All the media were autoclaved at 121 °C for 45 min. Precipitates of crushed bee brood were removed by sterile cotton before pouring to plates.

### 2.2. Growth Parameters

**Figure 5:**
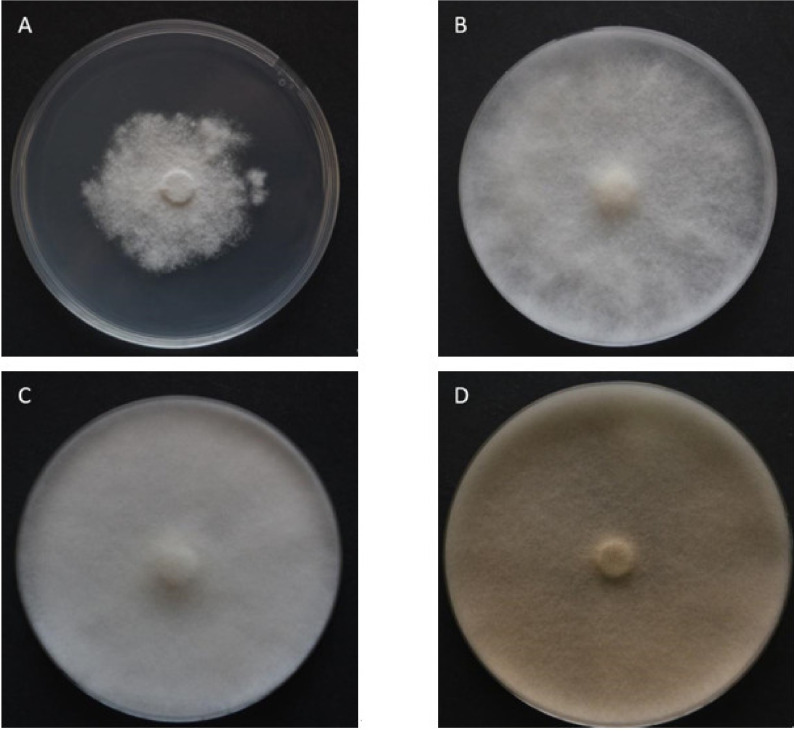
Growth differences among cultures of *A. apis* in different conditions. (**A**) Pure culture of (+) mating type of *A. apis* 15 days post inoculation on PDA at 35 °C (slow and irregular radial growth). (**B**) Pure culture of (+) mating type of *A. apis* 15 days post inoculation on PDA at 25 °C (moderate radial growth). (**C**) Pure culture of (+) mating type of *A. apis* 15 days post inoculation on PDA at 30 °C (fast radial growth). (**D**) Pure culture of (−) mating type of *A. apis* 15 days post inoculation on PDA at 30 °C (fastest radial growth and quick aging).

Seven-day-old cultures of each mating type were used in the experiments. The disks were cut from the edge of growing mycelium using an 8 mm diameter cork borer. One disk of the mating type (+) or mating type (−) was placed in the middle of each tested medium in Petri dishes (90 mm). All plates were placed into plastic bags to keep a stable humidity (>90%) and avoid water evaporation from the media. All the variants were incubated at different temperatures (25 °C, 30 °C, and 35 °C) for six days. Four replications of each variant were performed. Colony diameter was measured daily with a 1 mm precision rule by two perpendicular lines across the bottom of each Petri plate crossing over the inoculum plug until the mycelia reached the edge of plate (84 mm in diameter) or ceased growth (Figure 5).

### 2.3. Reproductive Structure Production

For the production of reproductive structures, six media (PDA, PDAY, SDA, MEA, YGPSA, and PDA-BB4) were selected based on the results of the previous experiment. One disk of mating type (+) and one disk of mating type (−) were placed symmetrically 5 cm apart from each other on all tested media. Four replications were prepared for each variant. All the variants were incubated at 25 °C, 30 °C, and 35 °C in plastic bags to keep a stable humidity (>90%). After 15 days of incubation, two disks (Ø 8 mm) were cut from the fully sporulated area at distances of 20 and 40 mm from the edge of each plate (a total of eight samples from each variant). Disks were separately transferred into a 1.5 mL microcentrifugation tube with 0.5 mL of 0.05% sterile water with Tween 80. The reproductive structures were washed out using a vortex for 60 s. The concentrations of cysts, spore balls, and ascospores from the obtained suspension were counted simultaneously in a Neubauer hemocytometer chamber (Sigma-Aldrich, Prague, Czech Republic) under a light microscope (Olympus CH20, Prague, Czech Republic) at 100× magnification (spore cysts and spore balls) and at 200× magnification (ascospores). The concentration of reproductive structures was recalculated for 1 mm^2^ of a fully sporulated area.

### 2.4. Morphometry of Reproductive Structures

The same suspensions prepared for counting of reproductive structures also were used for morphometry of spore cysts and spore ball assessment. An aliquot of 50 µL of the suspension was deposited on the surface of the glass slide and the reproductive structures were observed at 200× magnification under a light microscope (Nikon Eclipse E200, Prague, Czech Republic). Altogether, 15 cysts and 15 spore balls for each sample (120 cysts and 120 spore balls per variant) were measured from the captured images (Nikon, Prague, Czech Republic) by two perpendicular diameters with the software NIS Elements E200. The range of sizes was determined and the mean size for both structures was calculated for each variant.

### 2.5. Statistical Analysis

Three experimental designs with repeated measures on the Petri dish were used for exploring how *A. apis* grows and develops in response to several factors: (a) a 3 × 10 × 2 factorial design was applied to test the effect of temperature and medium on radial growth of two mating types on the fourth day; (b) a 3 × 6 × 3 factorial design was applied to test the effect of temperature and medium on the production of the reproductive structures (spore cysts, spore balls, and spores), and (c) a 3 × 6 × 2 factorial design was applied to test the effect of temperature and medium on the size of the reproductive structures (spore cysts and spore balls). Mating types, temperature, medium, reproductive structures, and their interaction were entered into the models as fixed effects, and Petri dishes were entered as random effects. For data analysis, repeated-measures nonparametric ANOVA was used, which is a robust statistical tool for the analysis of multiple factorial designs with non-normal residuals. Before using ANOVA itself, the data were transformed by the “art” function (ARTool package) [54]. This function first aligns the data for each effect (main or interaction) and then assigns averaged ranks [55]. The post hoc comparison of the main effect for food source was conducted by the “emmeans” package with Bonferroni-corrected *p*-values [56].

The radial colony growth was analyzed by the generalized estimating equation (GEE) approach with Poisson error distribution and log link function [57]. The repeated measures from the same Petri dish were partitioned within clusters by the argument “id” in the GEE method. Temporal autocorrelation between repeated measures was adjusted by specifying a first-order autocorrelation error covariance matrix. All analyses were performed using the statistical software R version 4.0.1 (R Core Team, 2020).

## 3. Results

**Figure 6:**
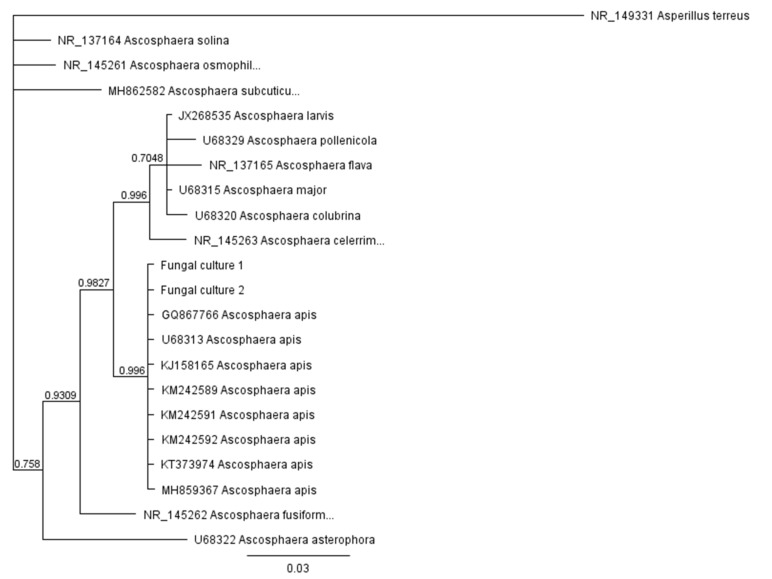
Phylogeny of the ITS region for selected *Ascosphaera species*. The phylogeny was inferred under Bayesian methodology in MrBayes using Geneious software. MrBayes was run for 100,000 generations with 50,000 sample points. For major phylogram branches, Bayesian posterior probabilities are shown. Taxa denoted as Fungal culture 1 and Fungal culture 2 represent sequence data generated in this study. These strains were isolated from mummified larvae, sequenced, and compared with other *Ascosphaera species*; their sequences as well as sequences for the outgroup (*Aspergillus terreus*) were obtained from GenBank.

We isolated two fungal strains from mummified honey bee larvae which were morphologically determined as *A. apis*. Sequencing of the ITS region was used for molecular characterization and determination of these two isolates. ITS sequences from both cultures possessed 100% sequence identity to ITS sequences from reference strains of *A. apis* (GQ867766, U68313, KJ158165, KM242589, KM242591, KM242592, KT373974, MH859367) as found by the BLAST analysis conducted on the NCBI website (https://www.ncbi.nlm.nih.gov/, accessed on 2 November 2020). Additionally, the result of phylogenetic analysis performed using Bayesian methodology in Geneious software clustered these stains into a single clade, along with eight reference *A. apis* strains (Figure 6).

### 3.1. Phylogeny of the ITS Region

We isolated two fungal strains from mummified honey bee larvae which were morphologically determined as *A. apis*. Sequencing of the ITS region was used for molecular characterization and determination of these two isolates. ITS sequences from both cultures possessed 100% sequence identity to ITS sequences from reference strains of *A. apis* as found by the BLAST analysis conducted on the NCBI website. Additionally, the result of phylogenetic analysis performed using Bayesian methodology in Geneious software clustered these stains into a single clade, along with eight reference *A. apis* strains (Figure 6).

### 3.2. Growth Parameters

**Figure 7:**
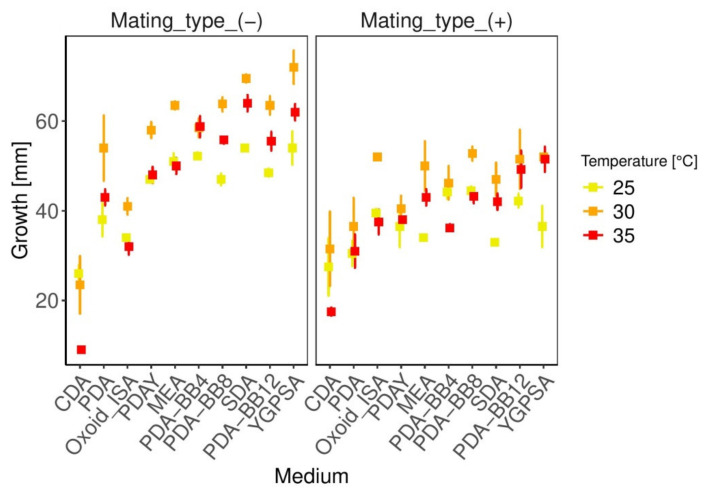
Effect of medium and temperature on the growth of both mating types of *A. apis* (4th day of growth). The error bars represent 95% confidence intervals.

**Figure 8:**
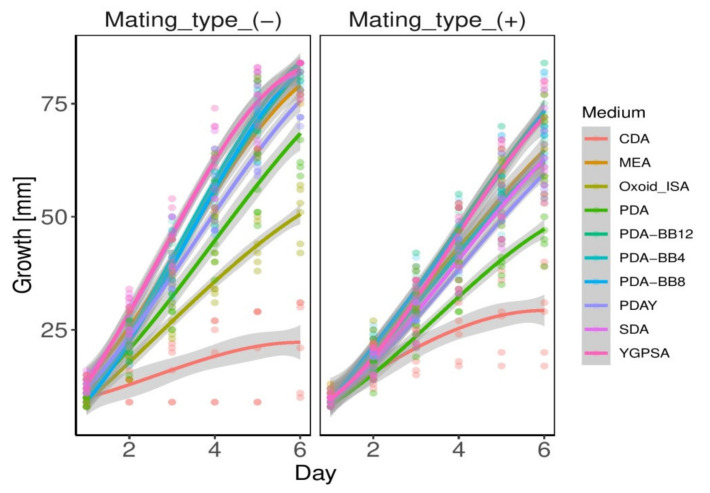
Scatter plot showing colony radial growth within six days, modeled using the cubic polynomial function. The regression lines were fitted by a generalized linear model with Poisson error distribution and link function log. The error lines represent 95% confidence intervals.

Both medium and temperature influence the growth rate of *A. apis* (Appendix A, Table A1, Figure 7). The growth is very close to the linear model (Appendix A, Table A2 and Table A3, Figure 8). Both mating types grew fastest at 30 °C on each artificial medium on the 4th day of growth, except the low-nutrient medium CDA (Figure 7), on which *A. apis* grew faster at 25 °C. Mating type (−) grew faster than mating type (+) on almost every artificial medium. The exceptions are the low-nutrient media such as CDA and Oxoid ISA (Figure 7). After six days of growth, the mating type (+) of *A. apis* did not reach the edge of any of the media plates at any temperature. The fastest growth of the (+) mating type was recorded on PDA-BB8 (Day 6: 25 °C—69.75 mm, 30 °C—80.75 mm, 35 °C—70.00 mm) and YGPSA (Day 6: 25 °C—58.00 mm, 30 °C—79.50 mm, 35 °C—79.00 mm). However, the mycelium of mating type (−) reached the edge of the Petri dishes in 11/30 different variations (25 °C—PDA-BB4, 30 °C—SDA, PDAY, MEA, YGPSA, PDA-BB4, PDA-BB8, PDA-BB12, 35 °C—SDA, YGPSA, PDA-BB4) in six days. The best artificial media for fast growth of both mating types are YGPSA, PDA-BB12, PDA-BB8, SDA, and PDA-BB4. MEA and PDAY are also very suitable media. On the contrary, the growth on medium CDA was insufficient. As the fungal cultures reached the edge of the Petri dishes on the fifth day, statistical analyses were performed with the data of the fourth day of growth.

### 3.3. Production of Reproductive Structures

**Figure 9:**
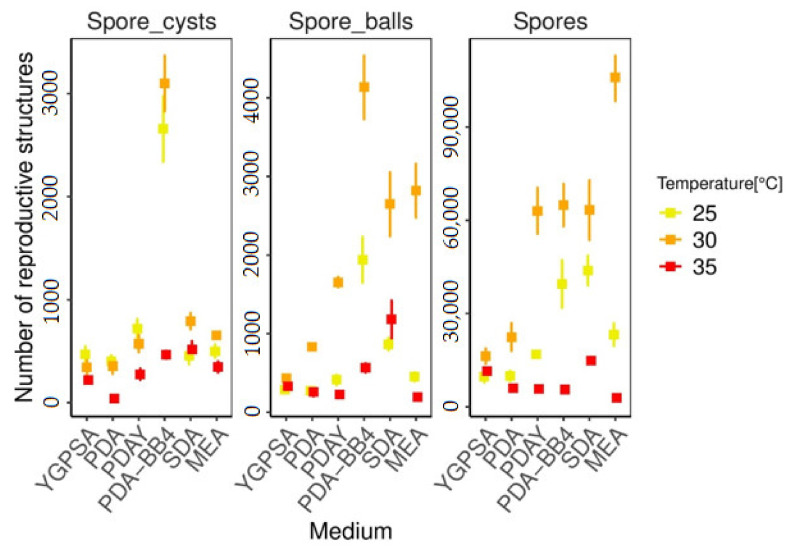
The mean number of reproductive structures (spore cysts, spore balls, and spores) per mm^2^ on different culture media in combination with temperature. The error bars represent 95% confidence intervals.

The number of reproductive structures, consisting of spore cysts, spore balls, and ascospores, was counted depending on the artificial media and temperature. Since ascospores are formed in spore balls and located in spore cysts, the reproductive structures were counted simultaneously to assess suitable conditions for the maturation and release of ascospores and spore balls. The production of reproductive structures is influenced by the artificial media and temperature (Appendix A, Table A4). The results are shown in Figure 9.

#### 3.3.1. Spore Cysts

For spore cyst production, both temperatures, 25 °C and 30 °C, are suitable. The temperature 35 °C is not suitable for high spore cyst production. The highest spore cyst production was recorded on the PDA-BB4 medium at 30 °C (3101/mm^2^), which was 4–9-fold higher than on the other media (30 °C—SDA: 792/mm^2^, MEA: 647/mm^2^, PDAY: 582/mm^2^, PDA: 356/mm^2^, YGPSA: 343/mm^2^). Thus, the addition of bee brood to an artificial medium has a significant effect on the fungus’s sexual reproduction.

#### 3.3.2. Spore Balls

Production of spore balls was also higher at 30 °C on all the tested media, especially on PDA-BB4 (4338/mm^2^), MEA (2822/mm^2^), and SDA (2645/mm^2^), which correlated with the number of spore cysts produced.

#### 3.3.3. Ascospores

Though the most ascospores were observed on MEA agar at 30 °C (99,245/mm^2^), the lowest number of ascospores was also produced on the MEA agar (2797/mm^2^), at 35 °C. Therefore, the appropriate temperature setting is crucial to ensure high ascospore production. Very high ascospore concentrations were also detected on PDA-BB4 (64,900/mm^2^), SDA (63,252/mm^2^), and PDA+Y (63,127/mm^2^), all at 30 °C.

### 3.4. Morphometry of Reproductive Structures

**Figure 10:**
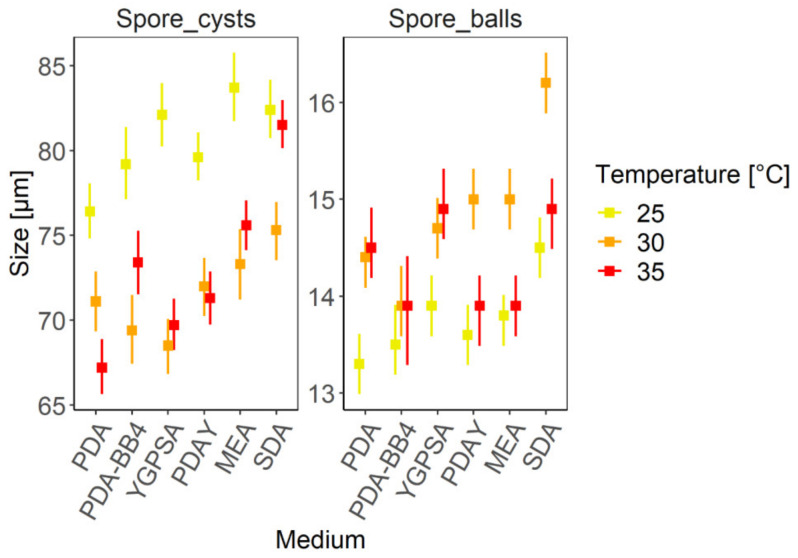
The mean size of reproductive structures (spore cysts and spore balls) in different culture media, in combination with temperature. The error bars represent 95% confidence intervals.

The temperature and artificial media affect the size of the reproductive structures of *A. apis* (Appendix A, Table A5). The results imply that, in more suitable media and temperatures, *A. apis* produce smaller spore cysts and bigger spore balls and vice versa (Figure 10). The biggest spore cysts were observed at 25 °C with the exception of SDA, where the result is not statistically significant. On average, the largest spore cysts were produced on SDA (25 °C—82.45 µm, 30 °C—75.28 µm, 35 °C—79.75 µm) and MEA (25 °C—83.75 µm, 30 °C—73.29 µm, 35 °C—77.54 µm). The smallest spore cysts were produced on PDA (25 °C—76.44 µm, 30 °C—71.08 µm, 35 °C—67.23 µm). This may also correlate with the amount of nutrients in the media.

The opposite situation is true of the spore ball size. Bigger spore balls were produced at a suitable temperature. If the temperature changed from the optimum, the size was smaller. On most of the tested media, spore balls were significantly bigger at 30 °C in comparison with 25 °C with the exception of PDA-BB4. In some cases (PDAY, MEA, SDA), the spore balls at 30 °C were bigger than at 35 °C. Overall, the largest spore balls were produced on SDA (30 °C = 16.21 ± 2.19 µm).

## 4. Discussion

### 4.1. Growth Parameters and Optimal Temperature

*Ascosphaera apis* grew faster on sugar-rich media such as SDA, YGPSA, MEA, PDA-Y, or PDA-BB. Slower growth was recorded on sugarless media such as CDA and Oxoid ISA (Figure 7). This is in agreement with Heath [58], who stated that *A. apis* grows very well on sugar-rich media because it can easily utilize arabinose, dextrose, mannose, galactose, sucrose, maltose, lactose, trehalose, glucose, fructose, and even dextrin and starch. On the contrary, high-fat and high-nitrogen media are not suitable for *A. apis* growth [59].

The optimal temperature for *A. apis* cultivation on commonly used artificial media was 30 °C for both mating types, (+) and (−). This could be one of the reasons why *A. apis* spreads faster in weak bee colonies, which are unable to maintain a high and stable temperature in beehives. Nevertheless, Flores et al. [37] reported the highest rate of infection in bee brood kept in vitro at 25 °C (77.62%), less at 30 °C (15.31%), and the lowest rate at 35 °C (2.22%). These differences between the growth of *A. apis* on artificial media and in natural conditions are probably caused by the low temperature for the development of bee brood. The lower than optimal temperature reduces the activity of physiological processes and contributes to a slower metabolism of bee brood, which makes bee brood more susceptible to pathogens [60,61].

Additionally, other studies reported an optimal *A. apis* growth rate near 30 °C [21,62,63]. However, *A. apis* is able to grow in a broad temperature range, from 22 °C [64] to 45 °C [65]. Therefore, the optimal growth conditions seem to depend on the particular strain and area of its occurrence.

Claussen [66] and Maurizio [63] stated that the male mating type (−) grows faster than the female mating type (+), which correlates with the results of this study. However, Evison et al. [67], Bissett [64], and Anderson and Gibson [62] did not notice any differences depending on the growth of opposite mating types. Claussen [66] and Bissett [64] also found yellowish pigment in the (−) mating type. The yellowish color was observed in this study as well; however, this phenomenon was not observed by Spiltoir [16]. Growth differences between opposite mating types probably depend on the particular *A. apis* strain, and the yellowish pigment could be caused by faster aging due to a lack of nutrients.

Since the male mating type (−) grows faster than the female mating type (+), it usually also requires more nutrients for its development due to a faster metabolism [68]. That is the reason for faster consumption of all nutrients from media and consequently the earlier aging and changing color. Low-nutrient media contain an insufficient amount of nutrients, so the generally faster-growing mating types are restricted. On the other hand, the slower-growing mating type can better exploit lower-nutrient media and, under those conditions, fares better (Figure 7).

Commercially available media can be improved by protein supplements. The addition of yeast to PDA medium promoted the growth of *A. apis*. Even better results were achieved by the addition of mixed bee brood to PDA (Figure 7, Appendix A, Table A3). The sufficient amount of bee brood to ensure faster fungal growth is 40 g/L media. In general, nutrient-rich media produced better results compared to the nutrient-poor media [47].

### 4.2. Reproductive Structure Production

To assess the reproductive structure development, the spore cysts, spore balls, and ascospores were counted simultaneously. The method does not allow for counting the real number of ascospores; instead, it involves assessing the natural level of maturation and release of spore balls and ascospores depending on the media and temperature. According to our results, both media and temperature affect ascospore release (Appendix A, Table A4), and thus a lot of immature ascospores can remain for different lengths of time in spore balls or cysts. Ruffinengo et al. [69] only counted ascospores after the stirring of the spore cysts. However, that method may be inaccurate because counting the real number of ascospores is very difficult as not every ascospore is properly released from reproductive structures.

In general, the more suitable the medium and temperature for *A. apis* development, the more ascospores and spore balls will mature and be released in a shorter period of time and vice versa. At 25 °C, more spore cysts than spore balls were observed on each medium (Figure 9), which indicated slow reproductive structure development and maturity because of the lack of ascospores and spore balls released. However, at 30 °C, significantly more ascospores and spore balls were released. The lowest reproductive structure release rate was recorded at 35 °C due to less favorable conditions. In addition, if there is less suitable medium, spore cysts crumble very little and only a small number of spore balls and ascospores are released. Ascospore maturation and release are heavily influenced by the temperature. For example, the lowest and highest ascospore concentrations were observed on MEA at 35 °C (2797/mm^2^) and 30 °C (99,245/mm^2^), respectively. However, since *A. apis* produced the greatest number of spore cysts and spore balls on the PDA-BB4 medium, this medium has the potential to produce the greatest number of ascospores as well. In this case, the ascospore maturation takes longer than on MEA agar, indicating that malt extract accelerates maturation. Nevertheless, Ruffinengo et al. [69] did not find a significant effect of the culture medium on the number of produced ascospores. This was likely due to the different media composition compared to this study.

In general, the highest spore yields are obtained in media with a C/N ratio of 10:1, which could represent potato dextrose agar (PDA) [50]. However, in this experiment, *A. apis* reproductive structure production was lower on PDA at the optimal temperature (30 °C) in comparison to the other tested media. These parameters were improved only after the addition of yeast (PDAY), but especially by honey bee brood supplementation (Figure 7, Figure 8 and Figure 9).

The number of produced ascospores can vary greatly, even under the same conditions. Ruffinengo et al. [69] reported only 7.42 ascospores per 1 mm^2^ on PDA at 30 °C, but we recorded 22,410 ascospores per 1 mm^2^ in the same conditions. The ascospore production could have been affected by a particular strain and also by the height of mycelium in the Petri dishes where the ascospores were produced. On most of our tested media, the mycelium grew to the top of the lid. Therefore, reproductive structures were produced in the whole column of mycelium. At the time of sample collection, most of the mycelia were degraded and the number of reproductive structures per area increased. The other issue is the area of the Petri dishes where the samples were taken from, because the sporulated area can differ in size. In some cases, the sporulated area can be thinner than the diameter of the cork borer. The bigger the cork borer that is used, the fewer reproductive structures are counted in the average 1 mm^2^ area. In this study, the sporulated area covered the whole cork borer diameter. That could be the main cause of the differences in the abovementioned results. Other authors reported that the mean value of ascospores on a single mummy varies between 10^4^ and 10^9^ depending on bee mummy color, which is affected by loads of spore cysts [70,71], which correlates with our observations.

### 4.3. Morphometry of Reproductive Structures

This study confirmed that temperature and artificial media affect the size of spore cysts and spore balls (ANOVA, F_(10, 228)_ = 7.5380, *p* < 0.001) (Appendix A, Table A5). Ascospores are almost unaffected by media and temperature [69] and too small to measure precisely. For that reason, ascospore morphometry was not included in this study.

During the measurement of spore cyst and spore ball size, a specific pattern was found. In less suitable conditions, large spore cysts and small spore balls were observed, and vice versa (Figure 10). This means that, for example, *A. apis* growing on SDA at 30 °C (suitable conditions) produced relatively small spore cysts and big spore balls. On the contrary, *A. apis* growing on PDA at 25 °C (less suitable conditions) produced relatively big spore cysts and small spore balls (Figure 10). The reason could be that, in suitable conditions, *A. apis* does not need to create big and strong cysts to protect ascospores, instead investing energy into creating bigger spore balls, which means more ascospores as a result. On the contrary, in worse conditions, it is preferable to create big and strong cysts, which help ascospores to survive an unfavorable period of time. This phenomenon can also be observed when compared with two other studies from Argentina. While the authors examined spore cysts in unfavorable conditions on solitary bees *Xylocopa augusti* [72], the average size of spore cysts was significantly bigger than the spore cysts produced under suitable conditions on artificial media [69]. In addition, the solitary bees’ brood is exposed to fluctuating temperatures, which could be the main reason for the greater spore cyst production. In former studies, smaller spore balls (12.5 µm, 12.0 µm, 12.1 µm) and bigger spore cysts (80.2 µm, 70.0 µm, 84.5 µm) were found at lower temperatures [62,64,73], while bigger spore balls (up to 16 µm) and smaller spore cysts (50–60 µm) were found at 30 °C [23]. The pattern can be seen in all the tested media in this study. On average, *A. apis* produced the smallest spore cysts and biggest spore balls at 30 °C. Only in the case of PDA were the spore cysts smallest at 35 °C, which indicates a more suitable cultivation temperature on that medium at 35 °C. If the sizes of spore cysts created at 30 °C and 35 °C are not significantly different, the optimal cultivation temperature seems to be between these values. The exceptions are spore balls produced on PDA-BB4, the size of which was not affected by the temperature. The addition of bee brood could simulate natural conditions and eliminate the effect of temperature on the spore ball morphometry, which is evident in artificial conditions.

However, the size of reproductive structures can also differ considerably depending on the strain. That is obvious when we compare the abovementioned Argentinian strain and the strain used in this study, cultivated under the same conditions (PDA at 30 °C). In addition, the size of the spore balls of the Argentinian strain was not significantly affected by the culture media. That is not in agreement with the results of this study (ANOVA, F_(5, 228)_ = 33.7521, *p* < 0.001). The sizes of the reproductive structures were very variable and depended on the temperature and artificial media. For example, on PDA at 35 °C, the mean spore cyst size was only 67.22 ± 12.17 µm; however, on MEA at 25 °C, the mean spore cyst size was 83.75 ± 15.22 µm. Spore balls are also affected by media and temperature. On PDA at 25 °C and SDA at 30 °C, the mean spore ball sizes were 13.32 ± 2.27 µm and 16.21 ± 2.19 µm, respectively.

### 4.4. Strain Competition

The growth rate of *Ascosphaera apis* mating types, also representing the degree of virulence, is very important because of the superinfection model of evolution in which strains do not cooperate but rather compete with each other [67]. If there is only one parasite invading a host, it can fully exploit the host nutrients because of an absence of competition [74]. However, if the host is attacked by more parasites, which is very common in nature, even with *A. apis* [75], it is very likely that less virulent parasites will be outcompeted or suppressed by the faster-growing ones. That leads to the selection of more virulent strains [76], assuming a high transmission ability [77]. Evison et al. [67] claim that more virulent *A. apis* strains may have an evolutionary advantage. They observed an increase in virulence after artificial co-infection by several strains in three generations. Some of the replicate lines of the parasite even disappeared. The model of *Ascosphaera* superinfection has also been observed by Klinger et al. [78], who tested three *Ascosphaera* species in a mixed infection. However, increasing virulence often leads to a decrease in parasite fitness because of faster host death and fewer nutrients [77]. It is likely that, after some time, only the most effectively host-utilized strains will remain in an area. The very high- or low-virulence strains will disappear [67].

An exception would be, for example, a heterothallic species, where two opposite mating types are needed to ensure reproduction. In this case, there may even be an advantage for a less virulent and less abundant mating type [79,80]. The evolutionary advantage of slower-growing mating types is better competitiveness in specific situations—for example, they can better exploit poor nutrient sources because of their slow growth. On the contrary, a more virulent mating type needs more nutrients to support its faster growth. It is demonstrated in this paper that only on poor-nutrient media (CDA and Oxoid ISA) did the (+) mating type grow faster than the (−) mating type (Figure 7). Another advantage lies in the low reproduction limitation because a faster-growing mating type is probably more abundant. That means it is easier to mate and survive in a given area.

Reproduction with a slower-growing mating type can explain the occurrence of white bee brood mummies, which are supposed to be without ascospores; however, Gochnauer and Margetts [71] found a few spore cysts on the surface of white mummies. This indicates the presence of both mating types in the mummy, but the faster-growing one may have prevailed and consumed all nutrients from the host. In general, a more virulent mating type prevails and outcompetes a slower one in a body cavity; therefore, there is no or little sporulation on the surface of the mummy. White mummies can occur often and in significant amounts. Gilliam [81] observed 35% white mummies in his experiment. This could be a way of reducing ascospore loads in infected beehives, ensuring balanced parasite‒host evolution.

## 5. Conclusions

The study provides a comprehensive summary of the cultivation conditions for culturing *A. apis* on artificial media and explains the biology and specific behavior pattern of the fungus depending on different conditions. The optimal temperature for the fungus cultivation is 30 °C because it allows the fastest growth, the highest reproductive structure production, the fastest release, and the greatest spore ball formation. The results of the study clarify the genesis of reproductive structures depending on the conditions and confirm the importance of the bee brood for the pathogen’s high reproduction rate. The article also gives a possible explanation of the occurrence of pure white chalkbrood mummies, which is based on the different growth rates of individual strains of *A. apis.* This mechanism balances the parasite–host relationship in nature. These results can be used in subsequent studies, which are needed to gain a better understanding of *A. apis* pathogenesis and virulence, especially in the conditions of a live bee brood.

## Data Availability

The study did not report any data.

## Appendix A

**Table A1:** Results of the aligned rank transformed ANOVA testing the effect of temperature, culture medium, and mating type on the radial growth of *A. apis* (4th day of growth).

Explanatory Variables	F	Df.	Df. Residual	*p*-Value
Medium	21.204	9	180	<0.001
Temperature	16.370	2	180	<0.001
Mating type	38.523	1	180	<0.001
Medium:Temperature	0.956	18	180	0.5121
Medium:Mating type	4.591	9	180	<0.001
Temperature:Mating type	0.096	2	180	0.9082
Medium:Temperature:Mating type	0.096	18	180	0.9982

**Table A2:** Results of generalized estimating equation approach, testing the effect of temperature, culture medium, day (repeated measurements in time), and mating type on the radial colony growth.

Explanatory Variables	Df.	Χ^2^	*p*-Value
Medium	9	374.1	<0.001
Day	1	6724.2	<0.001
Mating type	1	290.0	<0.001
Temperature	2	297.5	<0.001
Medium:Day	9	81.8	<0.001
Medium:Mating type	9	423.6	<0.001
Day:Mating type	1	2.7	0.098
Medium:Day:Mating type	9	14.0	0.122

**Table A3:** Regression slopes estimated by the generalized estimating equation approach, testing the relationship between *A. apis* growth on different culture media within six days; mating type and temperature were used in the model as covariates.

Medium	Regression Slopes	Standard Error	*p*-Value
YGPSA	2.53	0.06857	<0.001
PDA-BB12	2.49	0.06282	<0.001
PDA-BB8	2.48	0.06392	<0.001
SDA	2.47	0.06833	<0.001
PDA-BB4	2.45	0.05889	<0.001
MEA	2.45	0.06612	<0.001
PDAY	2.36	0.06224	<0.001
PDA	2.22	0.06724	<0.001
Oxoid ISA	2.23	0.07234	<0.001
CDA	1.66	0.08585	<0.001

**Table A4:** Results of the aligned rank transformed ANOVA, testing the effect of temperature and culture medium on the production of different reproductive structures per mm^2^.

Explanatory Variables	F	Df.	Df. Residual	*p*-Value
Medium	314.91	5	162	<0.001
Temperature	1128.41	2	162	<0.001
Rep. structures	1549.62	2	162	<0.001
Medium:Temperature	183.04	10	162	<0.001
Medium:Rep. structures	235.06	10	162	<0.001
Temperature:Rep. structures	1268.48	4	162	<0.001
Medium:Temperature:Rep. structures	124.21	20	162	<0.001

**Table A5:** Results of the aligned rank transformed ANOVA, testing the effect of temperature and culture medium on the size of different reproductive structures.

Explanatory Variables	F	Df.	Df. Residual	*p*-Value
Medium	33.7521	5	228	<0.001
Temperature	85.8862	2	228	<0.001
Rep. structures	6794.2763	1	228	<0.001
Medium:Temperature	6.0024	10	228	<0.001
Medium:Rep. structures	24.3856	5	228	<0.001
Temperature:Rep. structures	127.0338	2	228	<0.001
Medium:Temperature:Rep. structures	7.5380	10	228	<0.001
